# What Can Meal Observations Tell Us about Eating Behavior in Malnourished Children?

**DOI:** 10.3390/ijerph16122197

**Published:** 2019-06-21

**Authors:** Antonina N. Mutoro, Ada L. Garcia, Charlotte M. Wright

**Affiliations:** 1 Human Nutrition, School of Medicine, Dentistry and Nursing, College of Medical, Veterinary & Life Sciences, University of Glasgow, Glasgow G31 2ER, UK; Ada.Garcia@glasgow.ac.uk; 2 Child Health, School of Medicine, University of Glasgow, Glasgow G51 4TF, UK; charlotte.wright@glasgow.ac.uk

**Keywords:** responsive feeding, eating behavior, malnutrition, appetite, complementary feeding, malnutrition, children

## Abstract

Responsive feeding is an important aspect of child care, yet little is known about child eating and caregiver feeding behavior in Kenya. This study aimed to develop a mealtime observation methodology and assess child eating and caregiver feeding behavior in healthy and undernourished children in Nairobi. Healthy (*n* = 6) and undernourished (*n* = 13) children aged 6–24 months were observed during a meal, with standardized rating of child interest in food, mood, distraction and caregiver responsiveness. Eating and feeding behavior varied with the stage of the meal. Child interest in food decreased and child and caregiver distraction increased as the meal progressed. Healthy children were happy and interested in food during meals, but undernourished children often had low interest in food (7/13). The 7 undernourished children eating home food were distracted (3) and unhappy (5) but children eating ready-to-use therapeutic foods (6) were all happy and undistracted. Caregivers of healthy children offered encouragement more often during meals than caregivers of undernourished children (5/6 healthy, 3/13 undernourished). Meal observations were resource intensive and could give only a snapshot of the child feeding experience. More efficient research methods that can capture a general assessment of infant eating behavior are needed.

## 1. Introduction

Infant and young child feeding is a complex but important aspect of child care that affects child growth and survival. Inadequate feeding practices are associated with higher prevalence of undernutrition and related poor appetite [1,2]. Thus, a better understanding of child eating (act of consuming food) and caregiver feeding (act of giving food) behavior and the factors which influence them is required.

Ideally, children should be fed responsively, with caregivers responding directly to a child’s needs, while considering the child’s developmental level and ability [3]. However, lack of encouragement (positive active physical help and verbalization during meals) and force feeding occur regularly during meals in many low- and middle-income countries (LMIC), where caregivers are faced with challenges such as poverty and food insecurity [4,5,6,7,8,9,10,11]. In a meal observation study in Nicaragua, Engle and Zeitlin [5] used an active feeding and a child demand scale to assess eating and feeding behaviors (EFBs) in moderately undernourished children aged 12–19 months. They observed that caregivers only encouraged children to eat in 39% of feeding events observed and that food refusal occurred in 65% of events [5]. Similarly, a study assessing self-feeding, active feeding, responsive feeding and social interactions in moderately undernourished children aged 8–24 months in rural Bangladesh, showed that force feeding was common during meals and caregivers only encouraged children when they refused to eat [6]. In Vietnam, Ha and colleagues reported encouragement in only one-third of feeding events in children aged 12 and 18 months [10]. Although informative, most studies were carried out in rural or semi-rural areas [4,6,9,11,12,13,14]. Few observation studies have been carried out in urban informal settlements [5].

The aims of this study were to (1) observe meals in a low-income area in Nairobi, Kenya, where up to 40% of children are undernourished [15,16], in order to develop a standard mealtime assessment methodology for use in an urban slum setting, and (2) to describe how EFB varied with child nutrition status and the type of food offered. Findings from this study show that poor appetite and non-responsive feeding were common in the undernourished children. Although meal observations were informative, they were resource intensive and non-representative.

## 2. Materials and Methods

### 2.1. Ethics

The study protocol was reviewed and approved by the University of Glasgow College of Medical, Veterinary and Life Sciences (reference number: 200130125) and the Kenyatta National Hospital/University of Nairobi (P236/04/2014) ethics review committees, and approved by the National Council of Science, Technology and Innovation and the Ministry of Health (PMO/NRB/OPR/VOL1-3/35) in Nairobi. Approval to conduct the study at each of the facilities was also obtained from the relevant authorities.

### 2.2. Study Design and Setting

This was an exploratory observational study of children in their natural home environment. Meal observations were used to collect information on child eating and caregiver feeding behavior and interviews were used to collect socio-demographic data. Child caregiver pairs were recruited in August 2014 and July 2015 from one government (Mukuru health centre) and two faith-based health facilities (Ruben centre and Pipeline Presbyterian Church of East Africa—PCEA) in Pipeline and Mukuru slums in eastern Nairobi. Mukuru slum mainly comprises small semi-permanent structures made from iron sheet walls and roofs and cemented floors, while in Pipeline, housing mainly comprises one-room stone-built apartment buildings. Most residents work as casual laborers in the surrounding industries.

**Figure 1:**
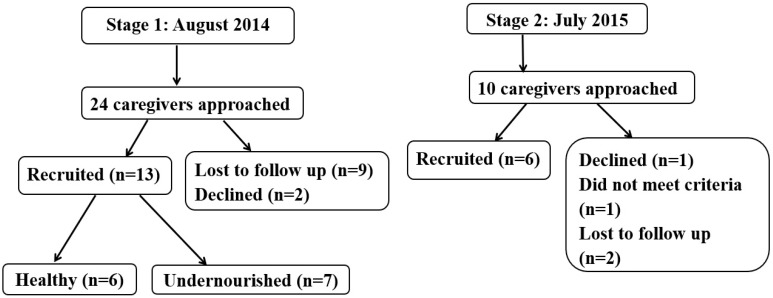
Participant recruitment flow. Only undernourished children were recruited at stage 2.

Purposive sampling was used to recruit caregivers of children aged 6–24 months attending child welfare clinics and outpatient therapeutic treatment centres (see Figure 1). During the first stage, healthy and undernourished children on a home diet were recruited from Mukuru health centre and Pipeline PCEA. During the second stage, undernourished children receiving ready-to-use therapeutic foods (RUTF), a fortified energy-dense peanut-based food, were recruited from Ruben centre. All meal observations were conducted in homes during the first stage, but during the second phase two observations were carried out in the health centre, due to security concerns.

### 2.3. Inclusion and Exclusion Criteria

Child–caregiver pairs were recruited if they were in the required age range, consented and were willing to set a date for an interview and supply an address. Children were excluded if they had illness or conditions that required specialized care such as edema, congenital disorders such as Down’s syndrome, or cerebral palsy.

### 2.4. Research Methods

Potential participants were identified by health workers, but data was collected by a doctoral student (A.N.M.). A detailed description of the study was provided to caregivers by A.N.M. and those who agreed to participate signed consent forms and provided a suitable date for a home visit. At this visit the researcher used a structured interview guide to collect information on socio-economic and demographic characteristics and feeding practices. Lunchtime meals and RUTF meals were then observed and a structured observation guide was used to record EFB. During observations, caregivers were encouraged to follow their usual feeding practices. The researcher also tried to position herself in a non-intrusive location, but this was not always possible because of limited space in the homes.

### 2.5. Structured Observation Measures

The meal observation schedule was informed by previous observation studies, which attempted to measure child appetite and caregiver responsiveness [6,10,17]. Unlike previous “all event” coding schemes [10,17] we created a recording schedule which rated behaviors at the beginning, 5 min into and at the end of the meal. Five min was selected because in other studies the average meal duration is usually 10 min [13]. Additional behaviors that occurred during meals and between the preset times were also recorded.

The observation schedule also recorded the time the meal started, location of the caregiver and child during meals, types of foods offered, and utensils used during the meal. A subjective assessment of the amount of food eaten was also collected, because other observation studies report that children rarely complete their meals [4,6]. For RUTF “meals”, the type of supplement offered, the prescribed dose and how the supplement was served (plain or mixed with other foods) was also included. The RUTF packet was classified as a “utensil” if the supplement was fed by squeezing from the packet directly into the child’s mouth [13].

### 2.6. Child Eating Behaviors

Four child behaviors were assessed: interest, mood, distraction and self-feeding. Interest in food is proxy measure of a child’s appetite as children who are not interested in food are likely to have a poor appetite. Mood enabled assessment of how cordial the meals were, and distraction enabled assessment of loss of a child’s attention. Self-feeding is an important aspect of young child feeding because it enables development of psychomotor skills and is associated with higher food acceptance [10]. A child was very interested in food if they readily opened their mouth and moved their head towards the spoon to receive food, neutral if they opened their mouth to receive food when food was offered but not eagerly, and not interested if they turned away from food every time they were offered food. Mood was defined on a 5-point scale ranging from excited to crying. Distraction during meals was noted where the child’s attention was diverted because they were playing with an object, playing with someone else or looking elsewhere. Self-feeding was defined as any bite a child fed themselves without assistance (see Table 1).

### 2.7. Caregiver’s Actions

Encouragement was assessed because caregiver encouragement during meals is associated with higher food acceptance [5]. It was defined as smiling at the child, praising the child, demonstrating to the child how to eat and lightly touching the child. Negative actions included flat verbalizations such as “eat your food”, threats or silence during the meal. Caregivers were distracted if their attention was diverted from the child during the meal. Behaviors were considered to occur all the time if they occurred continuously between each recording time.

### 2.8. Anthropometry

Weight, recumbent length and the mid-upper arm circumference (MUAC) were measured by the researcher using standardized procedures [18,19]. Weight was measured using a digital weighing scale (SECA 385 digital weighing scale III) to the nearest 0.1 kg. Length was measured to the nearest 0.1 cm using a portable length mat rollameter 100 (Raven Equipment Ltd., Dunmow, UK). MUAC was measured using a color-coded MUAC tape (S0145620 MUAC, Child 11.5 Red/PAC-50).

### 2.9. Analysis

Analyses were conducted using Statistical Package for the Social Science (SPSS) IBM Corp. Released 2010 Version 19.0. Armonk, NY: IBM Corp. Anthropometric information was converted to standard deviation scores using the World Health Organization 2006 growth standards. Children were classified as undernourished if they had weight, height or body mass index for age < 2−Standard Deviations.

Responses to the behavioral variables were recoded into binary responses as shown in Table 1. Child and caregiver actions were further summarized by counting the frequency of each action. The final eating and feeding behaviors were summarized into binary outcomes for easier interpretation. If an action occurred at least two times during the meal then it was scored as present and behaviors which occurred less than two times were scored as absent [6]. Statistical tests were not carried out because of the small sample size.

## 3. Results

Thirty-four caregivers were approached for meal observations, of whom 19 were recruited (Figure 1). The median (range) age of the caregivers was 27 (19 to 37) years; 18 resided in rented houses and 12 (5/6 healthy and 7/13 undernourished) resided in semi-permanent houses. Just over half the children (10/19) were female (Table 2). Healthy children were slightly older than undernourished children and undernourished children on RUTF had lower anthropometric measurements compared to those on home foods.

The foods offered were mainly carbohydrate-based with the few protein-rich sources mainly comprising legumes (Table 3). Most children (16) were fed by their mothers. One child was fed by a thirteen-year-old sibling, with the child’s mother present, and another by an aunt. One 15-month-old undernourished child was left to self-feed without assistance. All children were offered food from their own plates and caregivers either used spoons or their hands to feed children. Most children (15) were seated on the caregiver’s lap, with four seated on a chair, bed or on the floor. In only one case was the rest of the family also having their meal, with the child offered the same meal.

Child eating and caregiver feeding behavior at different stages of the meal are presented in Table 4 and Supplementary Figures S1–S3. Healthy children appeared to be more interested in food (Supplementary Figure S1a) and happy (Supplementary Figure S1b) during meals and their caregivers offered less encouragement (Supplementary Figure S2). In contrast, undernourished children were often not interested in food and their caregivers were more likely to offer encouragement. Undernourished children on home food were less likely to show low interest in food and were more likely to cry during meals than undernourished children on RUTF. Negative actions were common among undernourished children on RUTF (Supplementary Figure S3b). All four children on RUTF with complete observations had caregivers who showed negative actions. Compared to caregivers of healthy children, caregivers of undernourished children appeared to be more distracted, more so among undernourished children eating home foods.

Spitting out food was common during feeding among children on home foods (healthy 4; and undernourished 4). None of the undernourished children on RUTF spat out the supplement. Physical force during meals was observed during eight meals, mainly in the form of firm restraint of the child’s hands to restrict movement, and was more common in undernourished (6/13) than in healthy children (2/6). Only one caregiver, a 13-year-old sibling, attempted to force the child’s mouth open. Children who were restrained, tended to spit out food and turn away when food was offered. Other actions taken by caregivers included offering the child food again, talking about food to the child, calming the child down, questioning why the child was not eating and offering the child something else to eat. Two caregivers of healthy children talked to the child about food; one told the child the food is sweet and the other reassured the child to have only a small amount. None of the caregivers of undernourished children talked about the meal.

Promises to either give the child a sweet or take the child outside were common in children on home foods, especially in undernourished children on home diet (4/7). Two caregivers (one undernourished on home diet and one undernourished on RUTF) left the child alone when they refused to eat. Only one child, who was severely undernourished and aged 15 months, was left to self-feed without assistance from the caregivers; the community health worker in charge of this household was informed about this.

Healthy children were not breastfed during meals, but five undernourished children were breastfed before and during meals, 4/5 on home diet. Two children were offered the breast during a meal as a means of coaxing the child to open their mouth, two were breastfed after refusing home foods and one mother alternated between feeding RUTF and breastfeeding her child. Other foods and drinks offered when children refused to eat included porridge, milk and water.

## 4. Discussion

The aim of this study was to assess eating and feeding behavior using a standard mealtime assessment methodology that was feasible for use in an urban slum setting. A simple rating approach which relied on global assessments at three points during the meal was used rather than counting and timing predetermined actions, as has been done in other observational studies. The latter approach requires a much lengthier process of analysis and runs the risk that the pre-stated actions will not in fact be informative [17,20]. Because eating and feeding behaviors were recorded at specific time points, some behaviors were not systematically captured, but the researcher recorded additional actions as they occurred to provide a more comprehensive picture of the meal. Although multiple observations are ideal, they were not practical because of time constraints and security concerns, so that day-to-day variability in feeding practices and longer-term issues could not be captured.

Our findings suggested that eating and feeding behaviors varied with the stage of the meal, the child’s nutrition status and the type of food offered. This demonstrates the need to understand child eating patterns in order to maximize occasions when children readily accept food. Variations in child eating and caregiver feeding behavior by the type of food offered and the stage of the meal have previously been reported. In Nicaragua, Engle and Zeitlin [5] showed that interest in food was higher during snacks and bottle feeds than during midday meals, and children were more likely to refuse foods at the end of the meal [5].

Overall, healthy children showed more interest and were happier during meals. In contrast, undernourished children were less interested in food and less often happy when eating home foods. Low interest in food has been reported in previous observation studies in LMIC [4,5,6,12], especially among undernourished children, but direction of causation is still unclear [12]. Possible mechanisms which may have led to poor appetite in undernourished children in this setting include micronutrient deficiencies, underlying infections and chronic conditions such as environmental enteric disorder, all of which are likely to be common in urban informal settlements [21]. Whatever the causes of poor appetite, it appears to be relatively common among undernourished children, so a better understanding of this is required for successful management of undernutrition.

Interestingly, low interest in food was not seen when eating RUTF, suggesting that either children prefer RUTF to home foods or children on RUTF were recovering and regaining their appetite, but we did not ask how long the children had been receiving RUTF. Preference for RUTF over family foods has also been reported in Malawi, and was attributed to the taste and semi-solid consistency of the supplement [13]. High-energy ready-to-use foods are widely used for the management of severe and moderate acute malnutrition (MAM) and are usually offered for long periods of time. Considering the fact that these supplements are sweet and high energy, they are likely to lead to lower intake of complementary foods [22,23], especially when used for treatment of MAM, where treatment requires supplementation of the child’s regular diet with RUTF. More research is therefore required to assess the effect of RUTF on child appetite during and after treatment.

Young children appear to prefer liquid and semi-solid foods, which may explain the preference for RUTF and why caregivers opted to either breastfeed or offer drinks to their children when they refused to eat. Although breastmilk provides a significant amount of energy to children after the age of 6 months, its energy and nutrient content should ideally be complemented with energy and nutrient-dense foods. In cases where children are frequently offered breastmilk and low energy drinks, a feeling of satiety may be created at the expense of energy and nutrients, which may in turn have a detrimental effect on child recovery.

Caregivers generally tended to show negative behaviors and were less likely to show positive encouragement when feeding healthy children who were on average older. Low encouragement among healthy children is a possible indication that caregivers, in this setting, practice compensatory care, which is characterized by high levels of encouragement to compensate for poor child growth [5]. Low encouragement, the use of negative actions and force feeding are indications of non-responsive feeding, and appear to be common in LMIC [4,5,6,8,10,24]. This lack of responsivity may be attributed to food insecurity and poverty, competing demands on caregiver time, and cultural beliefs about feeding [25], but could also reflect a feeding style adapted to the child’s own temperament [26]. These feeding styles have the potential to negatively impact child growth, especially in cases where poor appetite is common [10,24]. There is therefore a need to further assess factors which influence caregiver behavior and also the impact of caregiver feeding behavior on child eating behavior.

Few previous meal observation studies have highlighted the challenges faced during observations, which creates a misleading impression that they are easy to conduct. It proved difficult to recruit participants and the process was time consuming. One meal observation was interrupted because fire broke out in a neighboring house and the researcher was threatened in the street by a disgruntled husband who did want his wife to participate. Gunshots were heard as the researcher made her way around the slums and on one occasion the researcher was almost robbed. Tracing participants back to their homes after recruitment was a challenge, because of the informal layout of the settlements; some did not have phones, while others provided wrong contact information. This meant that only the more cooperative participants could be studied. Although the meal duration itself was only 10 min in most cases, preparation for observations, locating households, interviewing mothers and taking anthropometric measurements usually took 2–3 h. All children were offered their midday meal at roughly the same time and therefore only one observation could be carried out in a day. Consequently, a small and potentially poorly representative sample of children were recruited. Findings from this study are therefore not generalizable or conclusive. This generally seems to have been true in previous studies, which have mainly been relatively small scale, with sample sizes ranging from 28 to 100 [5,6,10,13]. The meal observations described here were subsequently used to inform the design of an interview schedule, which allowed data on EFB to be collected from 415 caregivers in just a few months to provide a global picture of usual eating and feeding behavior over longer periods [27].

The presence of the researcher in the house may also alter the way the meal is offered, but participant reactivity is not usually assessed. There tends to be an assumption that behavior that is directly observed is more “true” than behavior that is reported by parents, although parents have far wider experience of their child. In a previous study in a more affluent setting, mealtime observations nested within a cohort study could not detect any difference in child behavior between weight faltering and heathy infants, yet parental questionnaires in the same cohort detected significant behavioral differences [17,28]. It is of note that further analysis of the same observational data did identify some differences in observed parental behavior [26].

## 5. Conclusions

Eating and feeding behaviors in this study appeared to vary with child nutrition status, type of food offered and the stage of the meal. Poor appetite and non-responsive feeding were common in undernourished children included in this study, especially among those eating home foods. Meal observations have the potential to provide important information about mealtime interactions but are resource intensive. A better understanding of factors which influence child eating behavior among undernourished children and how caregivers respond to it is required in order to develop sustainable programs of prevention and management of undernutrition in low and middle-income countries.

## Figures and Tables

**Table 1:** Coding and recoding of behaviors during meal.

Variable	Coding	Recoded
Child’s actions		
Interest	Very interested	Interested
	Moderately interested	
	Neutral	
	Less interested	Low interest
	Not at all interested	
Mood	Excited	Happy
	Very happy	
	Calm	
	Sad	Sad
	Crying	
Distracted	All the time	Distracted
	Most of the time	
	Sometimes	
	Rarely	Rarely distracted
	Not at all	
Caregiver’s actions		
Positive encouragement, negative actions, distracted	All the time	Sometimes
	Most of the time	
	Sometimes	
	Rarely	Rarely
	Not at all	

**Table 2:** Characteristics of children observed in different phases of the study. Data presented as median [range].

Characteristics	Healthy Home Food (*n* =6)	Undernourished Home Food (*n* = 7)	Undernourished RUTF (*n* = 6)
Age (months)	15.3 (6.6, 21.2)	12.6 (8.1, 20.1)	12.3 (9.9, 26.2)
Weight for age Z scores	−0.59 (−1.3, 0.11)	−2.59 (−5.75, −1.80)	−3.80 (−5.49, −2.74)
Length for age Z scores	−0.63 (−0.94, 0.87)	−1.90 (−6.41, 0.69)	−3.02 (−5.60, −1.61)
Weight for length Z Scores	−0.53 (−1.41, 0.09)	−2.31 (−3.81, −1.68)	−3.16 (−4.52, −1.51)
BMI Z scores	−0.56 (−1.36, 0.05)	−2.30 (−3.37, −1.35)	−3.09 (−4.54, −1.03)
MUAC Z score	−0.24 (−0.62, 1.39)	−2.11 (−4.37, −1.02)	−3.13 (−4.12, −1.21)
Meal duration (minutes)	12 (8, 25)	20 (10, 50)	14 (9, 21)

BMI: Body Mass Index; MUAC: Mid Upper Arm Circumference; RUTF: Ready to Use Therapeutic Foods.

**Table 3:** Foods offered during meal observations.

Child ID	Nutrition Status	Animal Protein	Other Protein Sources	Leafy Vegetables	Starch	Other Vegetables	Food Cooked with Oil
1	Undernourished	No	No	No	Pumpkin	Tomatoes	Yes
2	Undernourished	Milk	No	No	Ugali	No	No
3	Undernourished	No	No	No	Bananas and potatoes	No	No
4	Undernourished	No	No	Spinach	Pumpkin and bananas	Onions and tomatoes	Yes
5	Undernourished	No	Beans	Amaranth	Potatoes and bananas	No	No
6	Undernourished	No	No	Kale	Ugali	Tomatoes	Yes
7	Undernourished	No	No	No	Bananas and potatoes	Tomatoes	Yes
8	Healthy	No	Mung beans	No	Arrow roots	Onions and tomatoes	Yes
9	Healthy	No	No	No	Bananas and potatoes	No	No
10	Healthy	No	No	No	Pumpkin, potatoes, bananas	Onions and tomatoes	Yes
11	Healthy	No	No	No	Rice	Avocado, onions and tomatoes	Yes
12	Healthy	No	No	No	Bananas and potatoes	No	Yes
13	Healthy	No	No	No	Bananas	Avocado tomatoes onions	Yes

**Table 4:** Summary of child eating and caregiver feeding behavior during meals (*n* = 19).

Actions	Healthy (*n* = 6)		Undernourished Home Diet (*n* = 7)		Undernourished RUTF (*n* = 6) *	
	*n* %		*n* %		*n* %	
**Child Interest**						
Beginning	5	83%	3	43%	4	67%
Middle	5	83%	4	57%	3	75%
End	3	50%	2	29%	0	0
Present at least twice in in meal	6	100%	3	43%	3	75%
**Child mood (Happy)**						
Beginning	6	100%	4	57%	4	67%
Middle	6	100%	4	57%	4	100%
End	5	83%	2	29%	3	75%
Present at least twice in in meal	6	100%	2	29%	4	100%
**Child Distraction**						
Beginning	4	67%	3	43%	0	0
Middle	6	100%	4	57%	4	100%
End	4	67%	5	71%	1	25%
Present at least twice in in meal	4	67%	4	57%	0	0
**Caregiver positive actions**						
Encourage						
Beginning	1	17%	3	43%	1	17%
Middle	2	33%	2	29%	0	0
End	0	0%	1	14%	3	75%
Present at least twice in in meal	5	83%	2	29%	3	75%
**Caregiver Negative Actions**						
Beginning	4	67%	7	100%	6	100%
Middle	3	50%	6	86%	4	100%
End	5	83%	5	71%	2	50%
Present at least twice in in meal	4	67%	7	100%	4	100%
**Caregiver Distracted**						
Beginning	1	17%	4	57%	4	67%
Middle	2	33%	5	71%	2	50%
End	2	33%	5	71%	2	50%
Present at least twice in in meal	1	17%	5	71%	2	50%

* Only 4 undernourished children on Ready to Use Therapeutic Foods (RUTF) had complete meals because one child refused to eat and the other had already eaten part of their food.

## Supplementary Materials

The following are available online at https://www.mdpi.com/1660-4601/16/12/2197/s1, Figure S1: (a) Child interest in food (b) Child mood (c) Child distraction, by meal stage and nutritional status’ UN: Undernourished; Figure S2: (a) Caregiver encouragement (b) Caregiver negative actions (c) Caregiver distraction by meal stage and nutritional status. UN: Undernourished; Figure S3: (a) Child eating behavior and (b) Caregiver feeding behavior across whole meal, by nutrition status and type of food offered. Only 4 undernourished children on RUTF had complete meals because one child refused to eat and the other had already eaten part of their food UN Home: undernourished on home food; UN RUF: undernourished on ready to use foods.
