# Supplementary material for: What Can Meal Observations Tell Us about Eating Behavior in Malnourished Children?
Source: Int J Environ Res Public Health. 2019 Jun 21;16(12):2197. doi: 10.3390/ijerph16122197 (PMC6617361; doi:10.3390/ijerph16122197)
Supplement: Supplementary file 1 [file ijerph-16-02197-s001.zip › Supplementary figures/Figure 3.pdf]

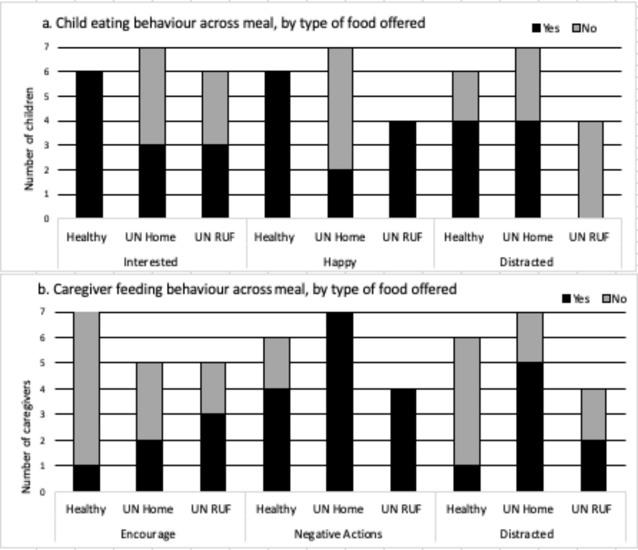
a. Child eating behaviour across meal, by type of food offered
Yes
No
Number of children
0
1
2
3
4
5
6
7
Healthy
UN Home
UN RUF
Interested
Happy
Distracted
b. Caregiver feeding behaviour across meal, by type of food offered
Number of caregivers
Encourage
Negative Actions
Distracted
