# Supplementary figures and images for: What Can Meal Observations Tell Us about Eating Behavior in Malnourished Children?
Source: Int J Environ Res Public Health. 2019 Jun 21;16(12):2197. doi: 10.3390/ijerph16122197 (PMC6617361; doi:10.3390/ijerph16122197)

### a. Caregiver encouragement

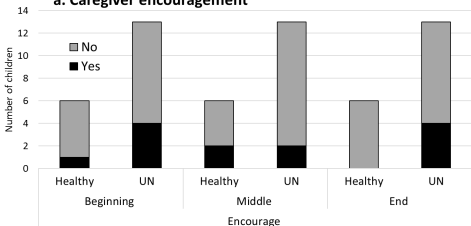

### b. Caregiver negative actions

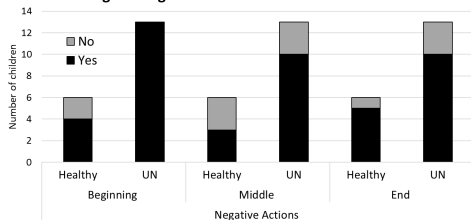

### c. Caregiver distraction

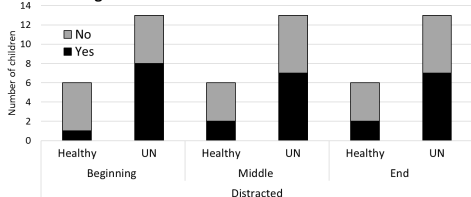

Supplement: Supplementary file 1 [file ijerph-16-02197-s001.zip › Supplementary figures/Figure 2.pdf]

### a. Interest in food

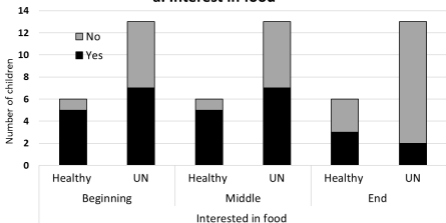

### b. Mood during meal

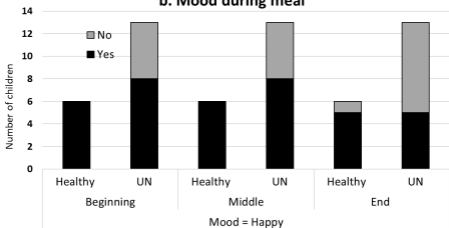

### c. Attention to meal

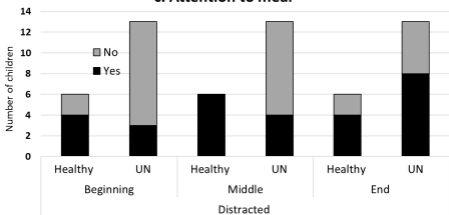

Supplement: Supplementary file 1 [file ijerph-16-02197-s001.zip › Supplementary figures/Figure 1.pdf]
